# Altered Transcript Levels of *MMP13* and *VIT* Genes in the Muscle and Connective Tissue of Pigs with Umbilical Hernia

**DOI:** 10.3390/genes14101903

**Published:** 2023-10-01

**Authors:** Jakub Wozniak, Weronika Loba, Alicja Wysocka, Stanislaw Dzimira, Przemyslaw Przadka, Marek Switonski, Joanna Nowacka-Woszuk

**Affiliations:** 1 Department of Genetics and Animal Breeding, Poznan University of Life Sciences, Wolynska 33, 60-637 Poznan, Poland; jakub.wozniak@up.poznan.pl (J.W.); weronika.loba@up.poznan.pl (W.L.); alicja.wysocka@igcz.poznan.pl (A.W.); marek.switonski@up.poznan.pl (M.S.); 2 Department of Pathology, Wroclaw University of Environmental and Life Sciences, C.K. Norwida 31, 50-375 Wroclaw, Poland; stanislaw.dzimira@upwr.edu.pl; 3 Department of Surgery, Wroclaw University of Environmental and Life Sciences, Plac Grunwaldzki 51, 50-366 Wroclaw, Poland; przemyslaw.przadka@upwr.edu.pl

**Keywords:** umbilical hernia, inguinal hernia, pig, DNA methylation, transcript level

## Abstract

Umbilical hernia (UH) and inguinal hernia (IH) are among the most common defects in pigs, affecting their welfare and resulting in economic losses. In this study, we aimed to verify the association of previously reported differences in transcript levels of the *ACAN*, *COL6A5*, *MMP13*, and *VIT* genes with the occurrence of UH and IH. We examined mRNA levels in muscle and connective tissue from 68 animals—34 affected by UH and 34 controls. In a second cohort, we examined inguinal channel samples from 46 pigs (in four groups). We determined DNA methylation levels in muscle tissue for the UH and control animals. The transcript level of *MMP13* changed in the UH cases, being upregulated and downregulated in muscle and connective tissue, respectively, and the *VIT* gene also showed an increased muscular mRNA level. The transcript of the *ACAN* gene significantly decreased in old pigs with IH. We further observed an increased DNA methylation level for one CpG site within the *MMP13* gene in UH individuals. We conclude that these alterations in gene mRNA levels in the UH animals depend on the tissue and can sometimes be a consequence of, not a cause of, the affected phenotype.

## 1. Introduction

Global pig production continues to grow with the increasing human population. Livestock provide many benefits such as employment growth, protein-rich food, and carbon storage [1], and the pig has become one of the most important livestock species [2]. The expanding pig industry needs to balance meat quality and animal welfare [3,4]. An important health issue in the pig industry is disorders like umbilical and inguinal hernia [5,6,7], which can result in economic losses.

Hernia is one of the most widespread condition in pigs; it involves the abdominal contents protruding and forming a subcutaneous bladder beneath the navel (umbilicus) [8], in the case of umbilical hernia (UH), or protruding via the inguinal canal in the case of inguinal hernia (IH) [9]. It can be affected by both genetic and environmental factors, such as incorrect cutting of the umbilical cord, high pressure in the abdomen, and infections and lesions of the body layers [7,10]. It is know that the occurrence of hernia ranges between 0.05% and 6.6%, depending on the breed and size of the studied cohorts. For example, the incidence of umbilical hernia in the Landrace and Pietrain breeds was reported to be 0.05% and 0.08%, respectively. For scrotal hernia, these estimations reached 2.4% for the Landrace and 6.6% for the Yorkshire breed [11,12,13]. The heritability of this disorder is rather low at approximately 0.3 [14]. Affected animals often show retarded growth and reduced feed efficiency [5]. They also suffer from pain, which increases morbidity and mortality [15]. Hernia is thus a severe problem for both breeders and veterinarians [16].

Although several studies have focused on identifying the genetic markers associated with hernia (reviewed, e.g., by [16,17]), the molecular mechanism responsible for this disorder in pigs remains poorly understood [5,8,15,18,19]. Recent studies have used transcriptome analysis to highlight several genes as strong candidates which correlated with the occurrence of umbilical hernia [6]. The researchers used RNA-seq technology for umbilical ring tissue fragments in five healthy and five umbilical hernia pigs, identifying 230 differentially expressed genes. Some of these genes (*ACAN*, *VIT*, *LGALS3*, *EPYC*, *CCBE1*, as well as genes from the *COL* and *MMP* families) have been taken as key contributors, since they are involved in the remodeling of the extracellular matrix as well as in resistance, production, and integrity of collagen [6]. A similar study concerning scrotal hernia (a form of inguinal hernia) was also performed for the inguinal canal tissue collected from four affected and four normal pigs. This research uncovered 703 differentially expressed genes, and the *MAP1CL3C*, *MYBPC1*, *SLC8A3*, *FGF1*, *BOK*, *DES*, *TPM2*, and *SLC25A4* genes were indicated as crucial in hernia pathogenesis. These genes are involved in important processes such as cell differentiation, muscular system regulation, and myofibril assembly [20].

DNA methylation is one of the regulatory mechanisms which has an influence on gene expression. The effect of this epigenetic modification may vary depending on the location of methylated cytosines and the level of methylation itself. It was already reported that cytosine methylation in gene body is connected with increased expression. On the other hand, the existence of methylated sites in 5′-flanking regions, rich in CpG dinucleotides (called CpG islands) is associated with gene silencing and repression of transcription [21]. Therefore, it is justified to search for changes in DNA methylation level as a potential mechanism for altered gene expression.

The aim of this study was to determine the transcript level of the *VIT* (Vitrin), *MMP13* (Matrix Metallopeptidase 13), *ACAN* (Aggrecan), and *COL6A5* (Collagen type VI α 5 chain) candidate genes in the muscle and connective tissues dissected from the umbilical ring of pigs with UH and in the muscle tissue of inguinal canal of pigs with IH, and to search for epigenetic mechanisms, such as DNA methylation changes, that are responsible for mRNA level alterations.

## 2. Materials and Methods

### 2.1. Ethics Statement

The samples in this project were collected during routine veterinary procedures. This is permitted under Polish law and does not require the approval of the local Bioethical Commission for Animal Care and Use in Poznan, Poland (13 January 2020).

### 2.2. Groups, Phenotypes, and Sample Collection

#### 2.2.1. Pigs with Umbilical Hernia

Sixty-eight piglets were derived from a cross between a DunBred (Landrace × Yorkshire) maternal hybrid line and Landrace or Duroc paternal lines. The animals all came from the same commercial breeding farm located in Wielkopolska Province, Poland. The animals were of both sexes and were aged 3–4 weeks. They were assigned into two groups: 34 animals were classified by veterinarians as umbilical hernia cases, while the control group (N = 34) was composed of healthy siblings (animals from the same litter as the hernia cases but showing no signs of hernia). This resulted in 34 full or half sibling pairs, so the affected animal always had a healthy sibling. Most of these pairs were born by different sows with the exception of 5 litters (delivered by 5 sows), where samples were collected from two hernia cases and two healthy controls. The animals were sacrificed by headshot, which was followed by collection of muscle and connective tissue samples separately from around the navel. Tissue samples were immediately frozen in dry ice for further analysis.

#### 2.2.2. Pigs with Inguinal Hernia

Forty-six unrelated male pigs were included in this part of the analysis. The animals were a cross between the Polish Large White and Polish Landraces and came from local breeding farms. The pigs were divided into four groups on the basis of veterinarian opinion: group G1 contained ten healthy young animals weighing 30–35 kg without any signs of hernia. The second group (G2) consisted of fifteen animals weighing 30–35 kg and with nonregressive inguinal hernias. G3 group contained eleven pigs weighing 30–35 kg with regressed IH (the hernia was at first clinically observed, but disappeared once the animal’s body weight exceeded 25 kg). The fourth group (G4) contained ten adult animals weighing 100–120 kg with IH observed throughout their lives. For the G2 and G3 animals, a fragment of the inguinal canal was collected during the delayed castration procedure, while the samples from G1 (at a weight of 30–35 kg) and G4 (at weight of 100–120 kg) were collected post-mortem from the slaughter house.

### 2.3. RNA Extraction and cDNA Synthesis for qPCR Assay

Total RNA extraction was performed using an RNeasy Fibrous Tissue Mini Kit (Qiagen, Germantown, MD, USA). The 30 mg of tissue from each sample was homogenized in buffer RTL supplied with the RNeasy Fibrous Tissue Mini Kit (Qiagen) using TissueLyser LT (Qiagen). The following steps were performed in line with the manufacturer’s instructions. The quantity and quality of total RNA were assessed by using a NanoDrop ND-2000 spectrophotometer (Thermo Scientific, Waltham, MA, USA) with 260/280 nm wavelength readings.

Around 1 µg of RNA from each sample was reverse-transcribed using a Transcriptor First Strand cDNA Synthesis Kit (Roche, Mannheim, Germany) in a total volume of 20 µL, following the manufacturer’s directions. The cDNA was subsequently evaluated with a NanoDrop ND-2000 spectrophotometer (Thermo Scientific, Waltham, MA, USA) and diluted with nuclease-free water to approximately 700 ng/µL.

### 2.4. Quantitative Real-Time PCR

The cDNA was used in real-time PCR reactions running on a LightCycler^®^ 480 II (Roche, Mannheim, Germany). We analyzed *ACAN*, *COL6A5*, *MMP13*, and *VIT* from both connective and muscle tissue of 34 healthy animals and 34 animals with umbilical hernia. The procedure was additionally performed on muscle tissue from the inguinal canal for healthy and inguinal hernia pigs (46 animals in total). Primers were designed using Primer3Plus software (version 2.4; https://primer3plus.com/cgi-bin/dev/primer3plus.cgi; accessed on 2 June 2021; Supplementary Material Table S1). The reactions for each gene were performed in duplicate using a LightCycler^®^ 480 SYBR Green I Master (Roche, Mannheim, Germany) in a total reaction mix of 10 μL per well, following the manufacturer’s instructions. The thermocycler program began with an initial denaturation cycle at 95 °C for 10 min, followed by 45 cycles including denaturation at 95 °C for 10 s, annealing at 60 °C for 5 s, and elongation at 72 °C for 5 s. Melting curve analysis was carried out after each amplification in order to verify product specificity. The relative expression values of each gene were computed on the basis of the standard curve method with a series of ten-fold dilutions of a DNA of known concentration (standards). The relative changes in expression level of genes were normalized against the level of reference (internal control) of the *H3F3A* (H3 histone, family 3A) and *PPIA* (Peptidyl-prolyl cis-trans isomerase A) genes [22], as described by [23].

### 2.5. Methylation Analysis

An in silico analysis was performed for the two differentially expressed genes, *MMP13* and *VIT*, for which statistically significant results were observed in the transcript level. The analysis with CpGPlot software (version EMBOSS 6.6.0; http://www.ebi.ac.uk/Tools/seqstats/emboss_cpgplot/; accessed on 17 November 2022) pointed to several CpG sites in the 5′ untranslated region (5′UTR) of the first exon of *MMP13* and in exon 12 of *VIT* (Supplementary Material Table S2).

The PyroMark Assay Design 2.0 software (Qiagen) was used to design primers for the pyrosequencing analysis (Supplementary Material Table S1). Thirty-four animals with umbilical hernia and 34 controls were analyzed in this way. The DNA was isolated from the muscle tissue (the amount of connective tissue was insufficient to perform this analysis) using a Genomic Mini Kit (A&A Biotechnology, Gdańsk, Poland). The quantity and quality of DNA was assessed with the use of a NanoDrop ND-2000 spectrophotometer (Thermo Scientific, Waltham, MA, USA) with 260/280 nm wavelength readings. Five hundred nanograms of DNA was bisulfite-converted using an EZ DNA Methylation-Gold kit (Zymo Research, Irvine, CA, USA). Additionally, nonmethylated controls were prepared using REPLI g Mini Kits (Qiagen), in line with the manufacturer’s instructions. The fully methylated controls were prepared from 500 ng of DNA using CpG methyltransferase (*M.SssI*, Thermo Fisher) with incubation for 3 h at 37 °C. Next, PCR was performed with a PyroMark PCR kit (Qiagen), following the manufacturer’s instructions. The PCR conditions were: initial denaturation at 95 °C for 15 min; 44 cycles of denaturation at 94 °C for 30 s, primer annealing at 56 °C for both genes, for 30 s and elongation at 72 °C for 30 s, followed by a final extension at 72 °C for 10 min. The negative control samples (without the DNA template) were also prepared for each reaction. PCR amplification results were checked on 1.5% agarose gel electrophoresis. The pyrosequencing reaction for the amplicons was carried out on a PyroMark Q48 Autoprep (Qiagen) system using PyroMark Q48 Advanced CpG Reagents (Qiagen), following the manufacturer’s recommended protocol. Finally, PyroMark Q48 Autoprep software (Qiagen) was used to calculate the percentage methylation for each of the CpG sites.

### 2.6. Statistical Analysis

Statistical analysis was carried out in the R statistical environment (version 4.1.2) [24]. The Shapiro–Wilk test was used to test the normality of the data using the “shapiro.test” function from the “stats” package (version 4.1.2). Subsequently, to compare mRNA results for pigs with UH and controls, a *p*-value was computed using the nonparametric, two-tailed Mann–Whitney U test. The same test was used to compare different DNA methylation levels between the UH and control animals. Each methylated cytosine was examined independently. The “wilcox.test” function was used to calculate *p*-values with parameter “paired = FALSE”. Moreover, adjustment using the Benjamini and Hochberg (1995) [25] procedure (FDR) was performed for the mRNA results using the “*p*.adjust” function. Finally, the “cor.test” function was applied to perform the Spearman’s rank correlation test to examine the significance of the correlation between the *MMP13* relative mRNA level and the CpG methylation level.

Four groups of animals with inguinal hernia were compared. Initially the Shapiro–Wilk test and the Kruskal–Wallis rank sum test were used (“kruskal.test” function from the “stats” package (version 4.1.2)). Subsequently, Dunn’s test for multiple comparisons with the FDR correction was chosen as a post hoc test, in order to identify exactly which groups differed, using the “dunnTest” function from the “FSA” package (version 0.9.3). For the performed tests, the statistical significance cutoff was set at *p* < 0.05.

## 3. Results

### 3.1. Relative mRNA Levels of the Studied Genes in the UH Pigs

**Figure 1:**
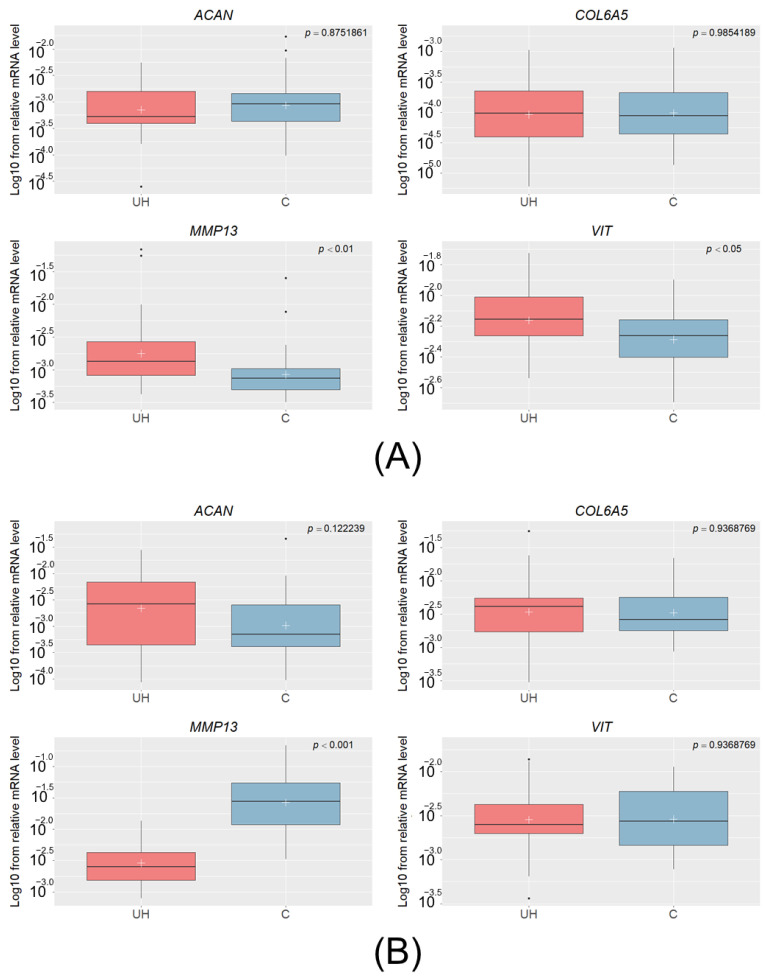
The Log10 of the relative mRNA levels of the *ACAN*, *COL6A5*, *MMP13*, and *VIT* genes in muscle (**A**) and in connective tissue (**B**) obtained from the UH (cases) and C (controls) animals. The white crosses denote mean values and the lines crossing the boxes represent medians. The lines under and over the rectangles denote the highest and lowest values, and the black dots under and over the boxes are outliers.

Quantitative real-time PCR was performed for *MMP13*, *VIT*, *ACAN*, and *COL6A5* in muscle and connective tissue for all 68 animals, revealing differences in the mRNA level of *MMP13* and *VIT* between the control and hernia pigs. The transcript level of *MMP13* was significantly higher in pigs with UH than in the muscle tissue of controls (*p_adj._* < 0.01); this was unlike the connective tissue, whose mRNA level was lower in the UH animals (*p_adj._* < 0.001). This indicates that the transcript level depends on the analyzed tissue. The only significant result for the *VIT* gene was observed in muscle tissue (*p_adj._* < 0.05), where a higher level of mRNA was found in UH pigs than in controls. For *ACAN* and *COL6A5*, the differences proved insignificant in all of the tissues (Figure 1).

### 3.2. Relative mRNA Levels of Studied Genes in IH Pigs

**Figure 2:**
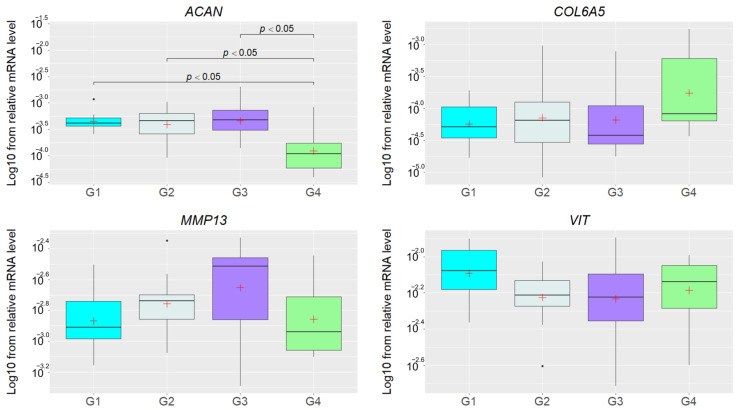
The Log10 of relative mRNA levels of the *ACAN*, *COL6A5*, *MMP13*, and *VIT* genes in groups of pigs without inguinal hernia and with different stages thereof. The white crosses denote mean values and the lines crossing the boxes represent medians. The lines under and over the rectangles denote the highest and lowest values, and the black dots under and over the boxes are outliers. The groups are indicated by G1, G2, G3, and G4.

The mRNA level was determined for *MMP13*, *VIT*, *ACAN* and *COL6A5*, but the Kruskal–Wallis test indicated significant results only for the *ACAN* (*p* = 0.02) gene. Subsequently, the post hoc test showed significantly lower transcript levels (*p_adj._* < 0.05) in group G4 than in groups G1, G2, and G3 (Figure 2), indicating that the altered *ACAN* expression was not related with the presence of hernia. On the other hand, we found that the expression of this gene was significantly downregulated in group G4 represented by adult IH pigs (>100 kg), so the observed differences can be related to the animal’s age.

### 3.3. CpG Methylation Level

**Figure 3:**
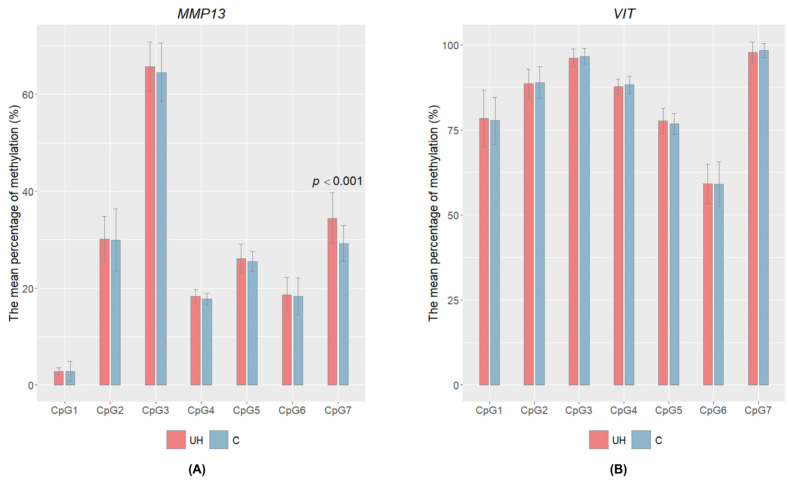
Mean percentage of DNA methylation ± SD in the CpG of two candidate genes: *MMP13* (**A**) and *VIT* (**B**) for UH and control pigs. The cytosines are indicated as CpG1, CpG2, CpG3, CpG4, CpG5, CpG6, and CpG7. Only one significant difference was observed for CpG7 in *MMP13*; the *p*-value refers only to this cytosine.

DNA methylation levels were examined in exon 12 for *VIT* and in the 5′UTR for *MMP13.* For both genes, cytosine methylation level was determined in the muscle tissue and seven CpG sites were studied for each gene (Supplementary Material Table S3). Only one statistically significant result was found for the single cytosine marked CpG7 in the *MMP13* gene (chr9: 33616509). In this case, we observed significant higher (*p* < 0.001) cytosine methylation levels in the UH animals (mean = 34%) than in the controls (mean = 29%); see Figure 3. However, there was no significant correlation between the *MMP13* relative mRNA level and the CpG methylation level at this position (ρ = 0.09342564; *p* = 0.4486). This observation shows that the DNA methylation of the studied region is probably not responsible for altered transcript level of *MMP13* gene.

## 4. Discussion

Information on the background of porcine hernia remains scarce [16]. Much effort has been put into identifying genetic variants that predispose to hernia development [5,6,8,15,18,19,26], but the polygenic nature of this condition has made such studies challenging. A new approach involving the transcriptome differences between hernia and control animals was thus undertaken for umbilical and inguinal hernia [6,20].

Here we studied the mRNA levels of four genes (*ACAN*, *COL6A5*, *MMP13*, and *VIT*) initially noted by Souza et al. [6] as strong candidates for hernia formation. The novelty of our study is that, in the case of umbilical hernia, we examined the muscle and connective tissues separately (dissected from the umbilical ring), which has not been done previously [6]. We also determined whether altered transcript levels in muscle tissue might be caused by differences in epigenetic mechanisms (DNA methylation). We additionally checked whether the same genes have altered expression in inguinal canal tissue collected from inguinal hernia pigs.

Our most interesting results were for the *MMP13* gene, in which the transcript level was increased in the muscle tissue and decreased in the connective tissue of the pigs with umbilical hernia. Expression of this gene has previously been associated with inguinal hernia in humans [27], while its downregulation has been noted in pigs with umbilical hernia by Souza et al. [6]. Those researchers, however, studied the transcriptome in a mix of different cell types collected from the umbilical ring. Here we were able to distinguish between muscle and connective tissues and found the opposite results. These differences may be caused by the different molecular function of the MMP13 protein depending on the expression site. MMP13 (known also as collagenase-3) is a member of the matrix metalloproteinase (MMP) family, which are proteins that degrade the components of the extracellular matrix and remodel connective tissue [28]. We can thus speculate that the reduced level of the *MMP13* gene in the connective tissue of the affected animals leads to incorrect tissue remodeling and possible lower tissue strength, which can predispose for hernia development. MMP13 also plays a role in wound healing [29], and it is known that cytokines and growth factors formed under pathological condition induce MMP levels [30]. The increased *MMP13* transcript level observed in the muscle tissue in our study might thus be rather a consequence of the hernia condition. It can be suggested that an elevated level of MMP13 is needed for wound healing (e.g., that the disruption of muscle tissue by the hernia induces increased repair processes). This hypothesis is supported by earlier studies, in which the involvement of the *MMP13* gene in tissue healing and repair processes has already been confirmed [31,32].

We also checked whether the differences in the mRNA level of the *MMP13* gene are the results of DNA methylation changes. Due to the limited quantity of connective tissue sample, this analysis was performed only for the muscle tissue. We found that in the studied 5′UTR region (exon 1) the methylation level for one cytosine (CpG7) was significantly increased in the affected animals. It is known that the 5′UTR region contains multiple regulatory elements that are important for the correct initiation of translation [33]. Moreover, a recent broad study of DNA methylation changes during human early embryo development concluded that, in the 5′UTR region (where the high CpG density was observed), the methylation level was low and negatively correlated with gene expression [34]. However, in our study, the significantly methylated cytosine in the 5′UTR of the *MMP13* gene did not correlate with the transcript level of the *MMP13* gene. We are thus unable to determine whether the difference we observed in DNA methylation has a significant relation to the hernia condition.

One interesting result concerned elevated transcript levels of the *VIT* gene in UH pigs. This observation did not confirm the result reported by Souza et al. [6], who examined a mix of tissues from the umbilical ring to compare 5 UH with 5 control pigs; they noted that the level of *VIT* expression was reduced. The *VIT* gene encodes an extracellular matrix (ECM) protein, which contains two von Willebrand A domains. It seems that the VIT protein is associated with migration and cell adhesion, like the majority of von Willebrand A domain-containing proteins [35,36]. Based on this information, we suggest that the observed overexpression of this gene in muscle tissue may be an effect of the repair mechanism acting in response to muscle damage by the hernia. Our study did not show altered transcript levels of the *ACAN* and *COL6A5* genes in the tissues of the UH pigs.

Interestingly, significant differences in the mRNA level of the *ACAN* gene were found in the IH pigs. Differences were observable between young (group G1, G2, and G3) and old (group G4) pigs, but not between those with inguinal hernia and those which were healthy. We thus assume that the reduced mRNA level of *ACAN* is an effect of aging. It is known that this protein (aggrecan) is a proteoglycan and is a component of the extracellular matrix which exists in form of proteoglycan aggregates. It plays an important role in the function of cartilaginous tissues [37], as well as in skeletal development through its involvement in the endochondral ossification process [38]. Our observation could thus be explained by the role of the ACAN protein in collagen production, with young pigs needing higher levels in the intense growth phase, whereas aggrecan in adults is involved in skeletal development. Moreover, ACAN is an important element of cartilage extracellular matrix that is reduced as aging of the organism proceeds [39]. The *ACAN* gene was the only gene that was found to be differently expressed in the pigs with inguinal hernia in this study, and no differences were observed for *MMP13*, *VIT*, or *COL6A5*.

## 5. Conclusions

Pigs with umbilical hernia showed differences in mRNA levels for the *MMP13* gene in muscle and connective tissue dissected from the umbilical ring as well as changes in the DNA methylation of a single cytosine located in the 5′UTR of *MMP13*. This may be the result of the demand for the MMP13 protein in UH pigs depending on tissue type. The *VIT* gene differed in transcript level in muscle tissues, which may be a result of umbilical hernia, rather than a cause. In the case of inguinal hernia, only the *ACAN* gene showed decreased mRNA levels in the muscle tissue of the inguinal canal in older animals, which may be a consequence of aging rather than pathological status.

## Data Availability

Not applicable.

## Supplementary Materials

The following supporting information can be downloaded from: https://www.mdpi.com/article/10.3390/genes14101903/s1, Table S1: primer sequences for the *ACAN*, *COL6A5*, *MMP13*, *VIT*, *H3F3A*, and *PPIA* genes used in qPCR and for the *MMP13* and *VIT* genes used in pyrosequencing; Table S2: CpG genomic position according to Sscrofa11.1. and their location in the gene context; Table S3: the mean percentage of DNA methylation with standard deviation and *p*-value in each studied cytosine, in two examined genes (*MMP13* and *VIT*), for both control and umbilical hernia groups.
