# Supplementary material for: Altered Transcript Levels of MMP13 and VIT Genes in the Muscle and Connective Tissue of Pigs with Umbilical Hernia
Source: Genes (Basel). 2023 Oct 1;14(10):1903. doi: 10.3390/genes14101903 (PMC10606093; doi:10.3390/genes14101903)
Supplement: Supplementary file 1 [file genes-14-01903-s001.zip › genes-2616974-supplementary.pdf]

Supplementary Material for: “Altered transcript levels of *MMP13* and *VIT* genes in the muscle and connective tissue of pigs with umbilical hernia”, by Jakub Wozniak et al.

Table S1. Primer sequences for the *ACAN*, *COL6A5*, *MMP13*, *VIT*, *H3F3A* and *PPIA* genes used in qPCR and for the *MMP13* and *VIT* genes used in pyrosequencing.

**Analysis**	**Target gene**	**Primer sequence**	**Amplicon size**
qPCR	*ACAN*	F: 5’ CAGGAGGGGTTGTGTTCCATTA 3’<br>R: 5’ CCTCCTCGAAAGTCAGTGAGTAG 3’	63 bp
	COL6A5	F: 5’ AGCTTTGGGCAGAACTTTGA 3’<br>R: 5’ TGCCAGAACTATCCACCAGA 3’	124 bp
	MMP13	F: 5’ AAGAGCATGGAGACTTCTACCC 3’<br>R: 5’ GGAGGAAAAGCATGAGCCAA 3’	64 bp
	VIT	F: 5’ GTCGAAGCCACCCACACTG 3’<br>R: 5’ AAGTCAGGTTCCTCCCCCA 3’	119bp
	H3F3A	F: 5’ CTTTGCAGGAGGCAAGTGAG 3’<br>R: 5’ TGGCATGGATAGCACACAGG 3’	72 bp
	PPIA	F: 5’ CACAAACGGTTCCCAGTTTT 3’<br>R: 5’ TGTCCACAGTCAGCAATGGT 3’	171 bp
pyrosequencing	*MMP13*	F: GGTTATGATGATTAAGTAAAGGTTAGT<br>R-biot*: TCCACCAACTCCAACAACTCT<br>Seq**: AGAGATAGTTTATAAAAATTTTGTG	318 bp
	VIT	F: GAGGTAGGTTGGAGGATGAGATTT<br>R-biot*:<br>TATCAAACTCCTTACTAAAATTAACCACAAA<br>Seq**: TTTGTAGTAAGATTTGTTTGAA	292 bp

PCR primers were designed based on pig genome sequence: Sscrofa11.1 (*ACAN* - GenBank ID: 397255, *COL6A5* - GenBank ID: 100515967, *MMP13* - GenBank ID: 397346, *VIT* - GenBank ID: 100524557). *reverse primer labeled with biotin; **sequencing primer

Table S2. CpG genomic position according to Sscrofa11.1. and their location in the gene context.

Gene	Cytosines	Chromosome position	Gene context
MMP13	CpG1	chr9: 33616595	5’UTR (exon 1, variant X2)
	CpG2	chr9: 33616586	5’UTR (exon 1, variant X2)
	CpG3	chr9: 33616563	5’UTR (exon 1, variant X2)
	CpG4	chr9: 33616557	5’UTR (exon 1, variant X2)
	CpG5	chr9: 33616546	5’UTR (exon 1, variant X2)
	CpG6	chr9: 33616521	5’UTR (exon 1, variant X2)
	CpG7	chr9: 33616509	5’UTR (exon 1, variant X2)
VIT	CpG1	chr3: 103384464	exon 12, variant X4
	CpG2	chr3: 103384458	exon 12, variant X4
	CpG3	chr3: 103384452	exon 12, variant X4
	CpG4	chr3: 103384446	exon 12, variant X4
	CpG5	chr3: 103384443	exon 12, variant X4
	CpG6	chr3: 103384431	exon 12, variant X4
	CpG7	chr3: 103384412	exon 12, variant X4

CpG positions were selected based on pig genome sequence: Sscrofa11.1 (*MMP13* - GenBank ID: 397346, *VIT* - GenBank ID: 100524557).

Table S3. The mean percentage of DNA methylation with standard deviation and p-value in each studied cytosine, in two examined genes (*MMP13* and *VIT*), for both control and umbilical hernia groups.

Gene	Cytosines	Umbilical hernia group		Control group		p-value
		Mean	Standard deviation	Mean	Standard deviation	
MMP13	CpG1	2.7941	0.7294	2.8235	2.0371	0.1593
	CpG2	30.1765	4.6546	29.9706	6.4172	0.6436
	CpG3	65.7059	5.1081	64.5294	6.0914	0.1477
	CpG4	18.3235	1.4296	17.7353	1.1886	0.0993
	CpG5	26.0882	2.9580	25.5000	2.0634	0.4793
	CpG6	18.6471	3.5665	18.3235	3.8433	0.7273
	CpG7	34.4118	5.2346	29.1765	3.7374	p<0.001
VIT	CpG1	78.4118	8.3016	77.7647	6.8625	0.4572
	CpG2	88.6177	4.2783	88.9412	4.6250	0.8578
	CpG3	96.2059	2.5320	96.6471	2.3469	0.5147
	CpG4	87.7059	2.2500	88.2353	2.5113	0.6896
	CpG5	77.7059	3.6889	76.7647	3.1533	0.2367
	CpG6	59.1765	5.7812	59.0882	6.5983	0.9706
	CpG7	97.7059	3.1482	98.3529	2.0727	0.7742
